# Impact of cannabis decriminalization on selected psychotic disorders: a retrospective analysis at “Center for Mental Health and Prevention of Addiction” (2013-2023) in Tbilisi

**DOI:** 10.1192/j.eurpsy.2025.2223

**Published:** 2025-08-26

**Authors:** N. Jibuti, L. Jishkariani, K. Jmukhadze, K. Gigolashvili, D. Zurabashvili, G. Lejava

**Affiliations:** 1 Tbilisi State Medical University; 2 Tbilisi Center for Mental Health and Prevention of Addiction; 3 Tbilisi Center for Mental Health and Prevention of Addiction; 4 Scientific-Expert Council on Psychiatry and Narcology, Tbilisi Center for Mental Health and Prevention of Addiction, Tbilisi, Georgia

## Abstract

**Introduction:**

Correlation between cannabis decriminalization and some psychotic disorders, specifically Acute and transient psychotic disorders (ATPD), Schizophrenia, and Psychotic disorder with delusions due to known physiological condition. It will cover overview of these disorders, challenges in differential diagnoses and the effects of drug abuse on mental health.

**Objectives:**

Impact of cannabis decriminalization on selected psychotic disorders: Acute and transient psychotic disorders (ATPD), Schizophrenia, and Psychotic disorder with delusions due to known physiological condition.

**Methods:**

- **Quantitative, Retrospective Analysis:** Analysis of archived medical records from 2013 to 2023 at “Center for Mental Health and Prevention of Addiction” in Tbilisi.
- **Examination of Prevalence Changes:** Focus on ATPD, Schizophrenia, and Psychotic Disorder with Delusions due to Known Physiological Condition.
- **Statistical Comparisons:** Comparison between pre-decriminalization (2013-2018) and post-decriminalization (2019-2023) periods.
- **Analysis of Drug User Patient Cases:** Assessment of the increase in drug user cases within the mentioned diagnoses.

**Results:**

- **ATPD Cases:** Increased significantly by threefold (from 195 to 594).
- **Schizophrenia Cases:** Decreased slightly by 21% (from 2068 to 1627).
- **Psychotic Disorder with Delusions due to Known Physiological Condition:** Remained relatively stable with a 14% decrease (from 473 to 408).
- **Drug User Patients:** Increased dramatically by about 3.5 times following cannabis decriminalization.

**Conclusions:**

- The findings suggest a potential link between increased substance use, including cannabis, and the rise in ATPD cases.
- Decriminalization may have indirectly influenced accessibility and use of various narcotics.
- The lack of public education about the potential mental health risks of narcotic use could be a contributing factor.
- The observed decrease in Schizophrenia and Psychotic Disorder with Delusions due to Known Physiological Condition cases might be due to other factors requiring further investigation.
- These findings highlight the need for comprehensive research on the long-term consequences of cannabis decriminalization on mental health.
- Targeted public health interventions and mental health support services are crucial to address the challenges of rising narcotic use in Georgia.

**Disclosure of Interest:**

None Declared

